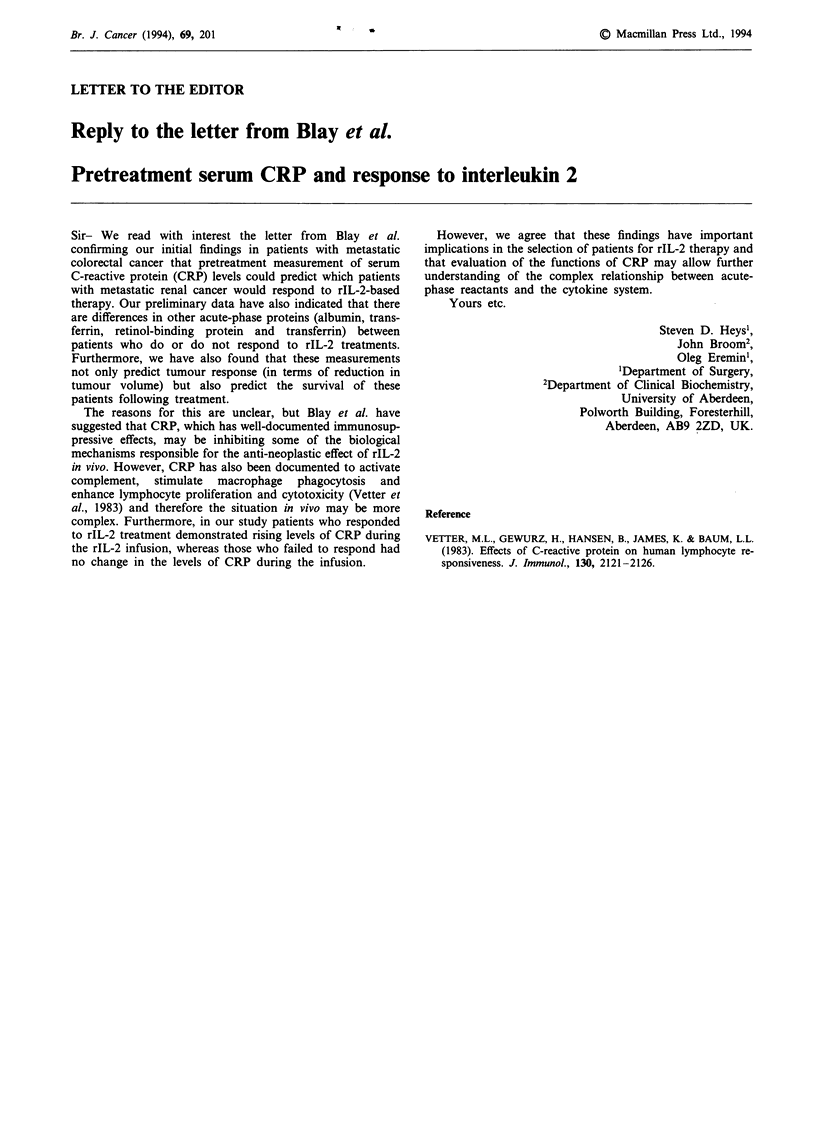# Reply to the letter from Blay et al

**Published:** 1994-01

**Authors:** Steven D. Heys, John Broom, Oleg Eremin


© Macmillan Press Ltd., 1994

LETTER TO THE EDITOR

### Reply to the letter from Blay *et al.*

### Pretreatment serum CRP and response to interleukin 2

Sir– We read with interest the letter from Blay *et al.* confirming our initial findings in patients with metastatic colorectal cancer that pretreatment measurement of serum C-reactive protein (CRP) levels could predict which patients with metastatic renal cancer would respond to rIL-2-based therapy. Our preliminary data have also indicated that there are differences in other acute-phase proteins (albumin, transferrin, retinol-binding protein and transferrin) between patients who do or do not respond to rIL-2 treatments. Furthermore, we have also found that these measurements not only predict tumour response (in terms of reduction in tumour volume) but also predict the survival of these patients following treatment.

The reasons for this are unclear, but Blay *et al.* have suggested that CRP, which has well-documented immunosuppressive effects, may be inhibiting some of the biological mechanisms responsible for the anti-neoplastic effect of rIL-2 *in vivo*. However, CRP has also been documented to activate complement, stimulate macrophage phagocytosis and enhance lymphocyte proliferation and cytotoxicity (Vetter *et al.*, 1983) and therefore the situation *in vivo* may be more complex. Furthermore, in our study patients who responded to rIL-2 treatment demonstrated rising levels of CRP during the rIL-2 infusion, whereas those who failed to respond had no change in the levels of CRP during the infusion.

However, we agree that these findings have important implications in the selection of patients for rIL-2 therapy and that evaluation of the functions of CRP may allow further understanding of the complex relationship between acute-phase reactants and the cytokine system.

Yours etc.

Steven D. Heys[1],
John Broom[2],
Oleg Eremin[1],
[1]Department of Surgery,
[2]Department of Clinical Biochemistry,
University of Aberdeen,
Polworth Building, Foresterhill,
Aberdeen, AB9 2ZD, UK.

### Reference

VETTER, M.L., GEWURZ, H., HANSEN, B., JAMES, K. & BAUM, L.L. (1983). Effects of C-reactive protein on human lymphocyte responsiveness. *J. Immunol.*, **130**, 2121–2126.